# Rabbit distal femoral critical size bone defect model: Vascular anatomy, surgical technique, peri-operative husbandry and biological response of empty and autograft-filled defects

**DOI:** 10.1371/journal.pone.0359512

**Published:** 2026-10-12

**Authors:** Daniel J. Wills, Rema A. Oliver, James D. Crowley, Michael J. Dan, Max J. Lloyd, Nat A. Puiu, David Jenkin, William R. Walsh

**Affiliations:** Surgical and Orthopaedic Research Laboratories, School of Clinical Medicine, UNSW Sydney, Sydney, New South Wales, Australia; Universidade de Trás-os-Montes e Alto Douro: Universidade de Tras-os-Montes e Alto Douro, PORTUGAL

## Abstract

**Introduction:**

Critical size bone defects do not heal spontaneously and are often used preclinically to evaluate the in vivo response to materials, devices or bone graft substitutes. Results may inform product development or satisfy regulatory body requirements, whilst also, providing clinically relevant information that cannot be obtained by in vitro or in silico experimentation. The New Zealand White (NZW) Rabbit distal femoral condylar defect in skeletally mature animals is a common model used in the development and evaluation of bone graft materials. When performed with appropriate consideration of the many pre, peri and post-operative factors the morbidity with this model is low. We present our experience with this model to provide a technical reference including the local vascular anatomy, surgical technique, peri-operative husbandry in large series between 2017–2022. The typical healing responses of empty and autogenous corticocancellous bone-filled defects is provided to assist in illustrating the model.

**Materials and methods:**

The local vascular anatomy was characterised ex vivo with anatomical dissection and intra-vascular injection of methylene blue, and an iohexol contrast study with micro-computed tomography. The in vivo animal model is described, highlighting the surgical approach, technique for defect creation and perioperative animal husbandry considerations. Examining outcomes from animals operated on over a 5-year period between 2017 and 2022 at our institution, the biological response following defect creation is also described qualitatively for empty defects (negative control) and autograft-filled defects (positive control) at 6 and 12-weeks post-operatively, with paraffin and polymethyl methacrylate (PMMA) histology and micro-computed tomography (µCT) endpoints.

**Results:**

2040 individual defects were created in 1020 rabbits confirmed to be skeletally mature with radiographs. Anaesthetic and intra-operative complications occurred with a very low incidence (1/ 1020 animals {0.1%}and 1/ 2040 defects {0.05%}, respectively). No post-operative complications impacting the healing outcomes or necessitating removal from the study occurred. Empty defects functioned as critically sized defects over a 12-week period, whilst autograft implanted into defects had incorporated into the surrounding cancellous architecture at 12 weeks. Closure of the cortical bone surface on the medial aspect of the femur was restored at 12 weeks, but not 6 weeks.

**Conclusion:**

In vivo models provide invaluable, irreplaceable preclinical data; however, such high-impact studies must have a sound ethical basis and be conducted with animal welfare and scientific rigour at the forefront of study design and execution.

## Introduction

Bone tissue is the second-most transplanted tissue after blood [1], with bone grafting procedures being performed in approximately half a million patients each year in the United States alone [2,3] and up to 2 million worldwide [4]. Bone grafts are used to augment the biological or mechanical healing environment in a variety of clinical treatment paradigms in orthopaedic trauma [2], spine [5], revision arthroplasty [6,7], dentistry [8] and veterinary orthopaedic disciplines [9,10]. Autogenous bone is often considered the “gold standard” graft material as it is available, low cost, poses no risk of disease transmission or tissue incompatibility and has a long track record of clinical success [4,5]. Harvesting this tissue however, creates additional donor-site morbidity in approximately 8–20% of cases [11,12]), with complications including seroma, haematoma, infection, intra or peri-operative fracture, hernia, chronic pain, scarring and poor cosmesis necessitating additional treatment with associated cost and healthcare burdens [12,13]. In addition, graft volume may be limited depending on defect size and harvest location and technique [14].

Certain limitations may be circumvented by the use of alternative graft options to augment or replace autogenous bone, however, the optimal substitute remains elusive. This highlights the need for robust preclinical testing of novel bone graft substitutes from both a scientific and regulatory perspective prior to clinical use [15]. In vivo critical size bone defect models provide an environment in which to evaluate the efficacy of an organic or synthetic material, device, or combination thereof.

A range of anatomical sites can be used to create defects to examine the healing response of graft materials including calvarial, cortical and cancellous bone. Critical sized bone defects, without surgical and/or medical intervention, are not expected to heal spontaneously over the lifetime of an animal [16–18]. When considering defects induced in the research setting, this definition can be modified to include defects which are not expected to heal spontaneously within the given time frame of the study. The rabbit is a well-established animal model for in vivo biomaterial testing due to its small size, docile nature and early skeletal maturity [19]. The distal femoral condyle (DFC) of the rabbit offers an optimal location for the creation of a critical size bone defect in cancellous bone, whilst maintaining an inherently mechanically stable environment without requiring supplemental fixation [20]. This model often forms a component of the Food and Drug Administration (FDA) 510 k submission regulatory approval process [21].

At our institution, cancellous defect creation and graft implantations are performed under surgical conditions simulating the clinical scenario whilst taking into consideration species-specific anatomical and biological differences. By emulating the clinical environment in the preclinical setting, we evaluate grafts materials in a robust experimental model, in conditions representative of those in which the material is intended to be used.

Preclinical animals models provide an important component of medical device research and scientific advancement. Standardization and uniformity across different studies would be advantageous for comparing data. In this paper we describe and share our experiences with this model including an ex vivo contrast media characterisation of the local vascular anatomy, surgical anatomical landmarks, surgical technique, model-specific peri-operative care considerations, typical experimental endpoints, and an interpretation of the typical healing response in empty defects and defects implanted with corticocancellous autograft, harvested from the iliac crest. This provides valuable qualitative data as well as species specific techniques for the design of future cancellous bone healing studies.

## Materials and methods

### Ex vivo vascular study method

Two pairs of hindlimbs were collected from freshly euthanised, skeletally mature, female, New Zealand White (NZW) Rabbits (*Oryctolagus cuniculus*), weighing over 3.5 kg at the conclusion of previously completed studies with institutional ethics approval (Animal Care and Ethics Committee, UNSW Sydney, Sydney). The hindlimbs were disarticulated at the coxofemoral joint.

Visual and radiographic characterisation of the vascular anatomy of the soft tissues overlying the surgical site was facilitated using contrast agents, 10% methylene blue and Iohexol (Omnipaque 300, GE Healthcare, Chicago IL), respectively. Iohexol was diluted in water for injection at a ratio of 1: 5 to create a 50 mg/ mL solution as is used for clinical intravenous contrast studies [22]. One limb from each pair (right) received methylene blue treatment and the other limb (left) Iohexol treatment. The skin was reflected anteriorly and removed to the level of the hock in all limbs. The femoral artery was isolated, transected at the level of the coxofemoral joint and circumferentially ligated to prevent escape of contrast solution. 2 mL of solution was instilled into the artery using a 27-gauge needle. The procedure was performed with the assistance of an operating microscope (Karl Storz, Tuttlingen, Germany).

Surgical dissection was performed through the deeper tissue layers in methylene blue infused limbs. Iohexol infused limbs were imaged using high-resolution radiographs (Faxitron, Tucson AZ) in orthogonal planes and micro-computed tomography (µCT). µCT images were obtained using an InveonTM micro-CT scanner (Siemens Medical Solution, Inc., USA). CT raw data was acquired using 80 kVp and 500 µa with 1250 ms exposure time, med-to-high-magnification, and binning of 2. Slice thickness was 54 µm and cylindrical scan length x diameter was 44 x 30 mm. Image reconstruction was performed in Inveon Acquisition Workplace (IAW client version 2.0, Siemens Medical Solution, Inc., USA). Following reconstruction, µCT images were manipulated and analysed in Inveon Research Workplace (IRW 4.2, Siemens Medical Solutions, USA).

### In vivo animal model

Records of skeletally mature, female, NZW Rabbits, weighing over 3.5 kg enrolled following institutional ethics approval (Animal Care and Ethics Committee [ACEC], UNSW Sydney, Sydney). All animals used in distal femoral defect models between 2017 and 2022 were reviewed and used to describe our technique and experience with the model. All animals treated with either empty or autograft-filled defects were reviewed to qualitatively describe the healing response.

We have previously reported comprehensive details of the perioperative animal husbandry for skeletally mature rabbits prepared for the single level posterolateral spinal fusion (Boden Model) [23]. The perioperative preparation for rabbits for the distal femur model is similar apart from the surgical site preparation and surgical procedure. Briefly, following delivery, the rabbits were individually weighed and housed in 3 − 3.2 m^2^ pens in groups of 3 – 4 on deep litter following institutional and legislative guidelines. Groups were observed closely and adjusted by relocating and re-socialising a dominant animal or multiple animals at 48 hours if unsatisfactory group hierarchy was noted. The rabbits were acclimated for at least 7 days prior to further handling.

Prior to study enrolment, animals older than 7 months of age underwent a veterinary physical examination to confirm physical health and suitability for study and screened under general anaesthesia for skeletal maturity using fluoroscopic imaging [23]. Radiofrequency identification microchips were implanted in the subcutaneous tissue between the shoulder blades of each animal for identification throughout the study.

#### Preoperative and anaesthetic considerations.

Premedication consisted of midazolam (5 mg/ mL at 0.3–0.5 mg/ kg) and buprenorphine (0.326 mg/ mL at 0.03–0.05 mg/ kg) intramuscularly using a 23 g needle. General anaesthesia was induced and maintained at a surgical depth by mask administration of isofluorane (2–3% to effect) and oxygen (100% at a rate of 2 L/ min). Fluid therapy consisting of a 20 mL/ kg bolus of warmed, balanced, isotonic fluid (Hartmann’s solution) was administered subcutaneously. Gel lubricant was instilled in the eyes throughout the procedure, and the animals were placed on a towel covered heat-mat. Physiological parameters including heart rate, respiratory rate, mucous membrane colour and perfusion, rectal temperature, presence of reflexes (palpebral, corneal, withdrawal), nociception, haemoglobin oxygen saturation percentage (SPO2%) and end-tidal carbon dioxide percentage (ETCO_2_) were constantly monitored by animal technicians with the assistance of a multiparameter cardiopulmonary monitor (Datalys V7, Lutech, Ronkonkoma, New York) throughout the procedures, and recorded every 10 minutes. Peri-operative antibiotic prophylaxis was provided in the form of procaine penicillin, 50000 IU/ kg via alternating subcutaneous injection sites every 48 hours, starting 24 hours prior to surgery and finishing 6 days post-operatively.

Blood for routine haematology and serum biochemistry screening was collected by cephalic or lateral saphenous venipuncture with a 22 g needle and 3 mL syringe and placed in ethylenediaminetetraacetic acid (EDTA) and serum tubes (BD Vacutainer®, Oakville ON), forming part of the systemic health assessment of each animal preoperatively and at the conclusion of the study. The blood work is reviewed to assess general health status of the animals as well as to provide baseline for comparisons depending on the study design and implanted materials.

#### Surgical preparation and approach.

In a dedicated animal preparation room, animals were positioned in dorsal recumbency. The hair was clipped circumferentially from the level of the coxofemoral joint proximally to the hock joint distally, with a # 50 clipper blade (Heiniger Opal, Switzerland). The skin was aseptically prepared with a combination of 2% chlorhexidine scrub and 70% ethanol wipes in several alternating applications, followed by a final spray of 4% povidone-iodine. The animal was covered by a large drape secured with towel clamps and fenestrations were created directly over the stifle joints as shown in Fig 1. Prior to defect creation, if indicated by the study design, corticocancellous autograft was harvested from the iliac crest, as previously reported [23] by surgical approach of the ilium and harvesting with rongeurs.

**Fig 1:**
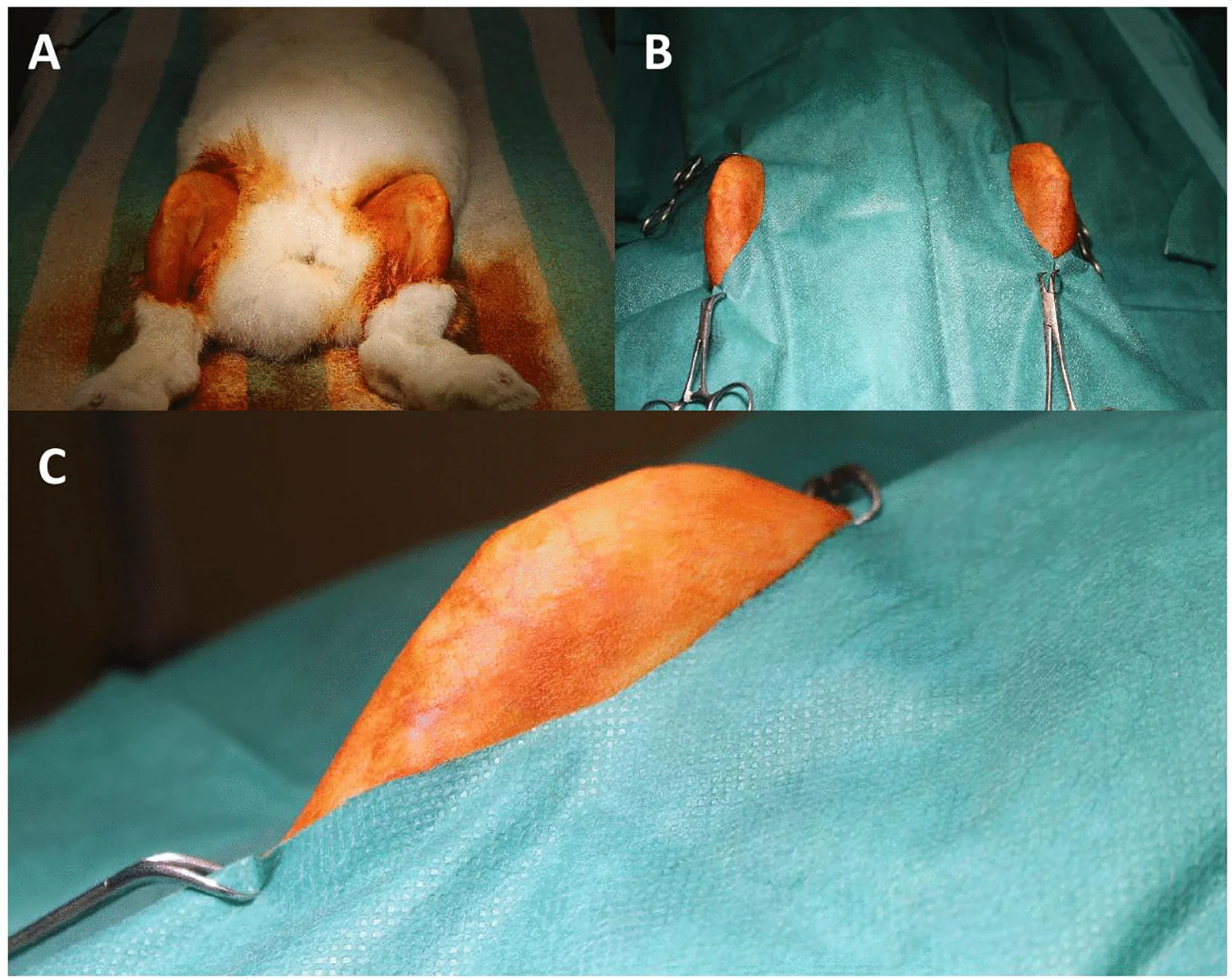
Patient positioning and surgical preparation. A: Hair clipped and skin aseptically prepared, animal placed in dorsal recumbency. B: Large drape placed over animal, secured in place with towel clamps and fenestrations made over each stifle. C: Medial aspect of contralateral stifle, opposite the surgeon’s seated position.

The surgical approach was achieved using a modest inventory of surgical instruments. Size 15 scalpel blade mounted on a number 3 handle, Freer periosteal elevator, thumb forceps, tissue scissors and needle driver facilitated the soft-tissue component of the procedure.

We found it most ergonomic to operate in a seated position and approach the contralateral limb, e.g., seated on the rabbit’s left side, approaching the medial aspect of the right limb. Orientation of the skin incision was aided by palpation of the patella cranially, the tibial tuberosity distally and the medial femoral and tibial condyles caudally. A 1 cm skin incision was made, extending from the caudal edge of the medial femoral condyle to the patella tendon along the caudal margin of the vastus medialis obliquus [24], oriented at an oblique angle of approximately 30° to the patella tendon. The incision was continued along the same plane through the underlying joint capsule. A Freer elevator was useful to help elevate the capsule from the surface of the bone and palpate anatomical landmarks for accurate defect placement (Fig 2).

**Fig 2:**
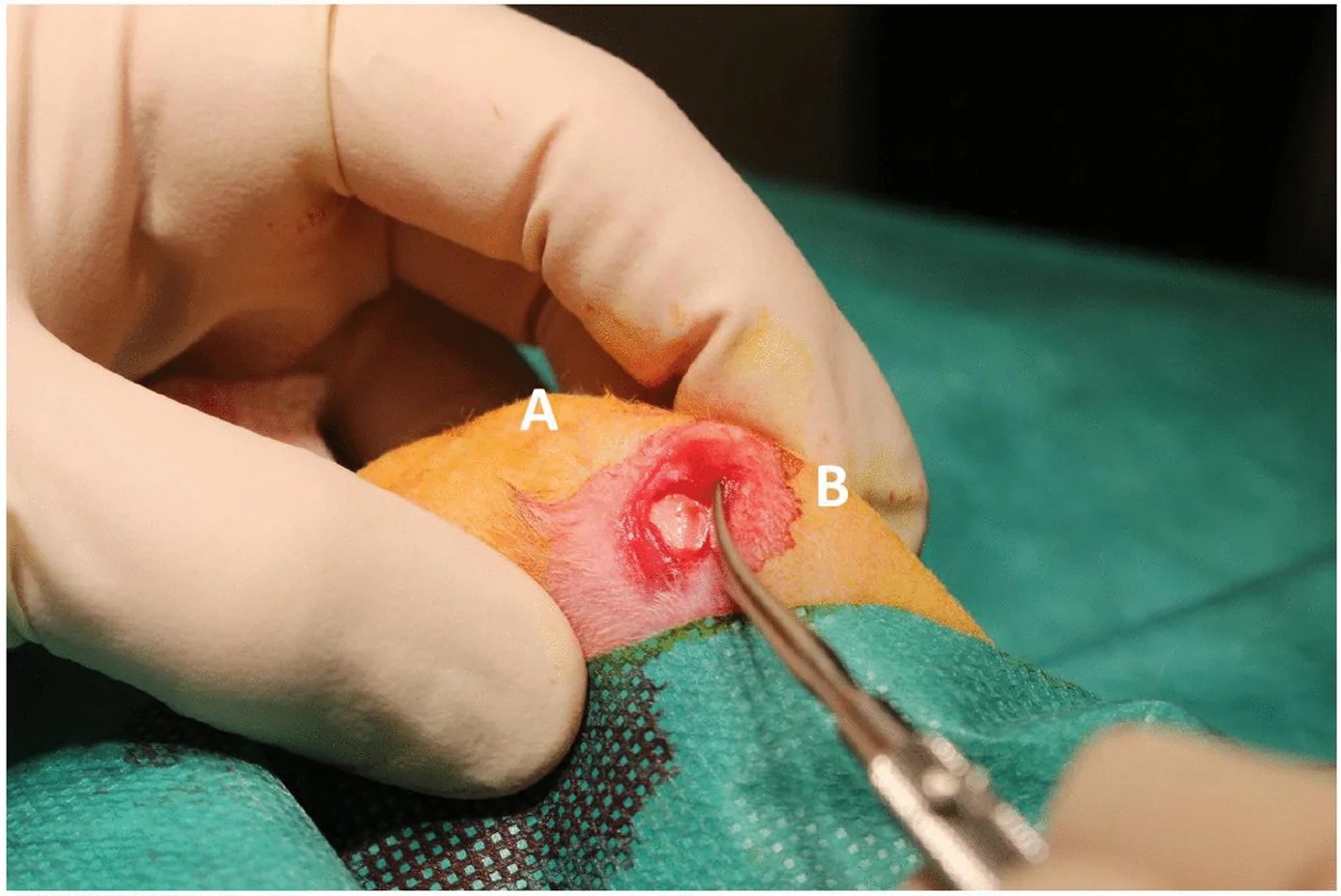
A Freer periosteal elevator was useful for mobilising overlying joint capsule and establishing access to the surface of the bone at the intended defect site. The elevator is positioned here at the femorotibial joint line. A: Patella, B: Tibial tuberosity.

Care was taken to identify and avoid large blood vessels in each tissue plane, however, if bleeding was encountered, it was readily controlled by applying firm pressure with a sterile cotton gauze swab for short period of time without the use of any electrocautery. The Freer periosteal elevator was then used to elevate the muscle belly, joint capsule and periosteum anteriorly, exposing the medial surface of the femoral condyle and the origin of the medial collateral ligament as an anatomical reference point for placement of the defect.

#### Defect technique.

For creation of the bony defect, a step-drilling/milling procedure was utilised to prepare a 6 mm diameter, 10 mm depth, cylindrical bone defect (Fig 3).

**Fig 3:**
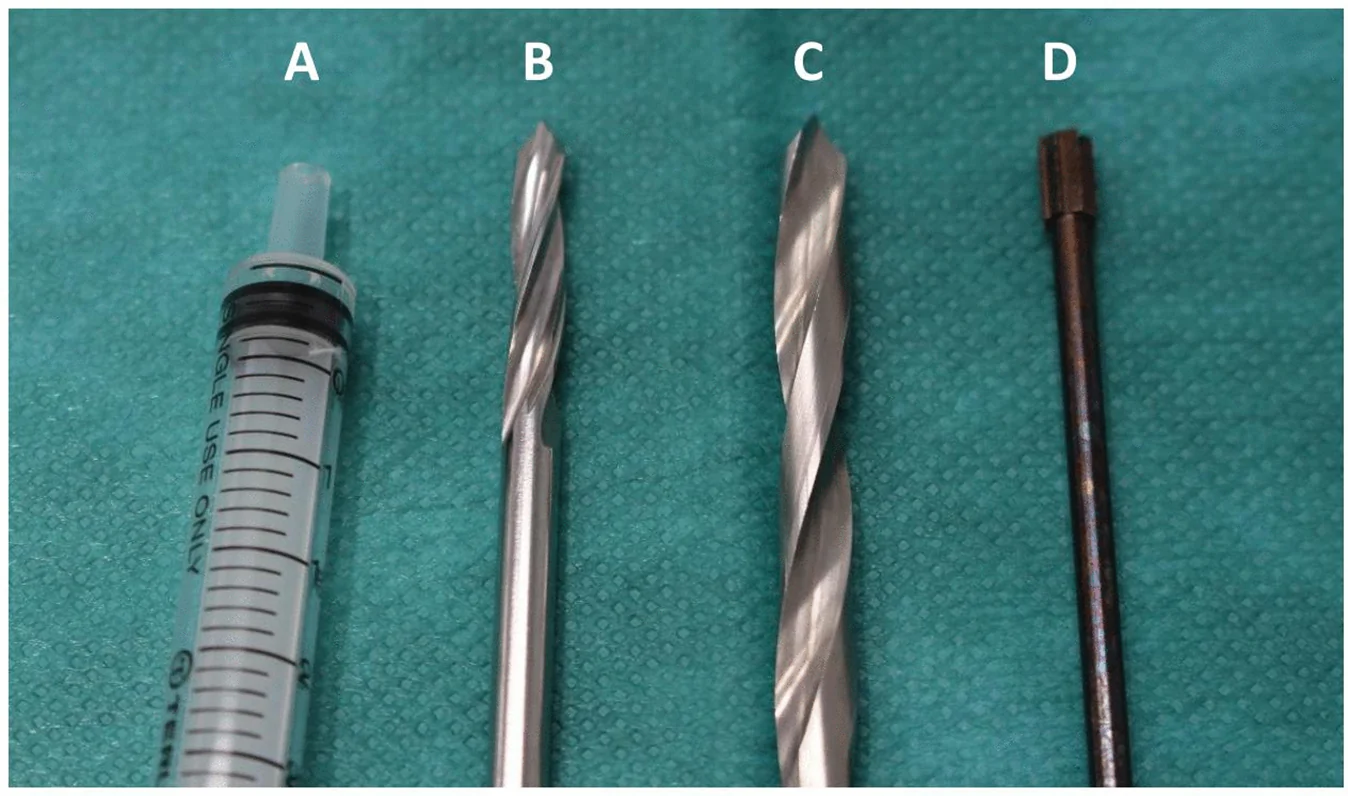
Instruments used in step-drilling/milling procedure for cylindrical bone defect creation. A: 3 mL syringe used by the surgical assistant to deliver external saline irrigation during drilling; B: 4.5 mm 3 – fluted, trochar – tipped drill bit (Surgibit®, Orthopaedic Innovation PTY LTD, Collaroy, NSW); C: 6 mm, 2 – fluted drill bit (Synthes, P.A. Paoli); D: MR8 fluted cylinder mill (Medtronic, Minneapolis, MN 55432).

We used a hand-held, pneumatic orthopaedic drill (Hall MicroAire®, CONMED, Utica, NY) with a maximum rotation speed of 700 RPM and no torque limit, with a regulated pressure of approximately 100 PSI. A 4.5 mm, 3-fluted, pyramidal tip stainless-steel drill bit (Surgibit®, Orthopaedic Innovation PTY LTD, Collaroy, NSW) was used to establish the starting point and trajectory of the defect. Following drilling to the intended depth, the 4.5 mm hole was enlarged to 6 mm with a standard 2 – fluted drill bit (Synthes, P.A. Paoli). The deep conical aspect of the defect created by the trochar point of the 6 mm drill bit was flattened using an MR8 fluted cylinder mill (Medtronic, Minneapolis, MN 55432). Saline irrigation was applied manually to effect by a sterile surgical assistant during drilling to minimise any thermal damage due to drilling (Fig 4).

**Fig 4:**
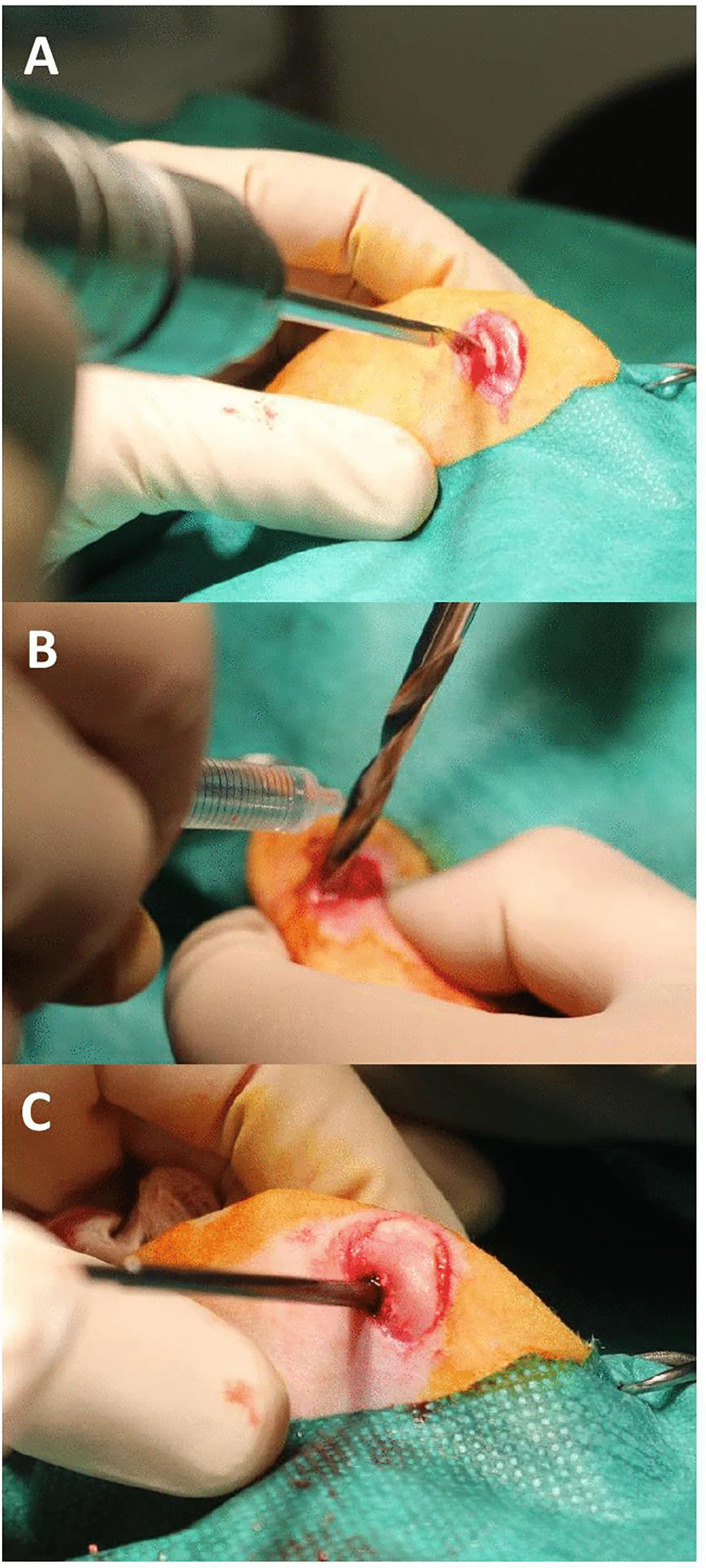
Drilling and milling steps used in creation of a rabbit distal femoral defect in the right hindlimb. (Head to the right of the image). A: 4.5 mm drill, B: 6 mm drill, C: 6 mm MR8 fluted cylinder mill. Saline irrigation is applied manually throughout the process by the surgical assistant as in image B.

#### Implantation of autograft.

Defects were lavaged with sterile fluid (e.g., 0.9% NaCl) to remove residual bone swarf and sterile cotton gauze swab was packed into the defect if required to control bleeding from the bone bed. Defects were either left empty or filled to the level of the cortical shell with corticocancellous autograft harvested from the iliac crest immediately prior to defect creation.

#### Surgical site closure.

The joint capsule and fascial layer were apposed over the defect using 3−0 braided absorbable suture (Dexon, Davis & Geck, North Ryde, NSW) in a simple continuous pattern, cutting suture ends 1 mm above the knot. The subcutaneous layer was closed similarly if sufficiently present, however, when minimal tissue was present, closure of the skin layer alone in a simple continuous pattern using 4−0 braided absorbable suture (Vilet, Riverpoint Medical, Portland, Oregon) resulted in good tissue apposition, minimal tissue dead-space and provided sufficient holding strength. Waterproof adhesive film (Opsite, Smith & Nephew) was applied to the skin to limit bacterial migration into the wound.

#### Post-operative care.

Animals were recovered between warm towels placed on top of a heat mat and monitored by dedicated staff. Anaesthetic recovery was swift, with most animals returning to ambulation within 30 minutes of the procedure.

Post-operative analgesia was provided by subcutaneous injection of non-steroidal anti-inflammatory drug (NSAID) (Carprofen at 4 mg/ kg or Meloxicam 0.5–1 mg/ kg) administration for a minimum of 3 days post-operatively.

Following recovery, animals were returned to the same group pens and monitored closely each day for the first 7 days for signs of pain, distress, systemic illness, or surgical complication by trained staff and veterinary team. The surgical sites were closely observed for evidence of excessive swelling, fluid discharge, heat, discolouration, or wound breakdown by trained staff. Animal handling was minimised during the acute monitoring period. Additional analgesia was administered in response to detection of signs of pain including inappetence, lethargy, reduced food and/or water intake, reduced faecal output, weight loss, altered mentation, behaviour or reduced ambulation or increased grimace score.

Long-term monitoring by dedicated animal care and veterinary staff was continued through to the conclusion of the study, including daily observations of external appearance, food and water intake, excretions, ambulation, behaviour and weekly close examination and bodyweight recording.

#### Euthanasia, harvesting of specimens and typical endpoints.

Animals were euthanised post-operatively by mask induction of isoflurane anaesthesia followed by intravenous injection of sodium pentobarbitone (Virbac Australia Pty Ltd, Milperra, Australia) at 1 mL/ 2.0 kg to effect. Following anaesthetic induction and prior to administration of pentobarbitone, blood was collected as described earlier.

Necropsy was performed immediately following death, with photographic documentation of the visual appearance of the surgical site, overlying skin underlying soft tissues and bone surface, as well as internal organs including heart, lungs, liver, spleen, kidneys, and popliteal lymph nodes. Internal organs were placed in cassettes, fixed in buffered 10% formalin prior to processing for paraffin histology. Femurs were removed by disarticulation of the coxofemoral and femorotibial joints, denuded of soft tissue and wrapped in 7.4% phosphate buffered saline-soaked swabs in preparation for radiographic imaging.

Endpoints used to evaluate the in vivo response include high-resolution radiographs (Faxitron), micro-computed tomography (μCT), paraffin and polymethyl methacrylate (PMMA) histology. High-resolution radiographs allow comparison to clinically used X-ray images, whilst µCT was used to evaluate the three-dimensional microstructure and subjectively assess presence of bone in the defects. Paraffin slides were stained with haematoxylin & eosin (H&E) and tetrachrome, whilst PMMA slides were stained with methylene blue and fuchsin. The cellular characteristics, tissue architecture and new bone formation were evaluated.

## Results

### Ex vivo vascular study results

An arterial branch coursed anterodistal from the trifurcation of the femoral artery at the level of the distal femoral diaphysis, previously identified as the articular branch of arteria suprema genu [25] along which arose multiple small branches to supply the surrounding musculature in a cranial and proximal direction, notably the caudal border of the vastus medius obliquus. This vessel, shown in Fig 5 with the local surgical anatomy, may act as a landmark at the proximal margin of the incision through the fascia and underlying joint capsule for access to the surface of the distal femoral condyle, or act as a source of haemorrhage during dissection in this region.

**Fig 5:**
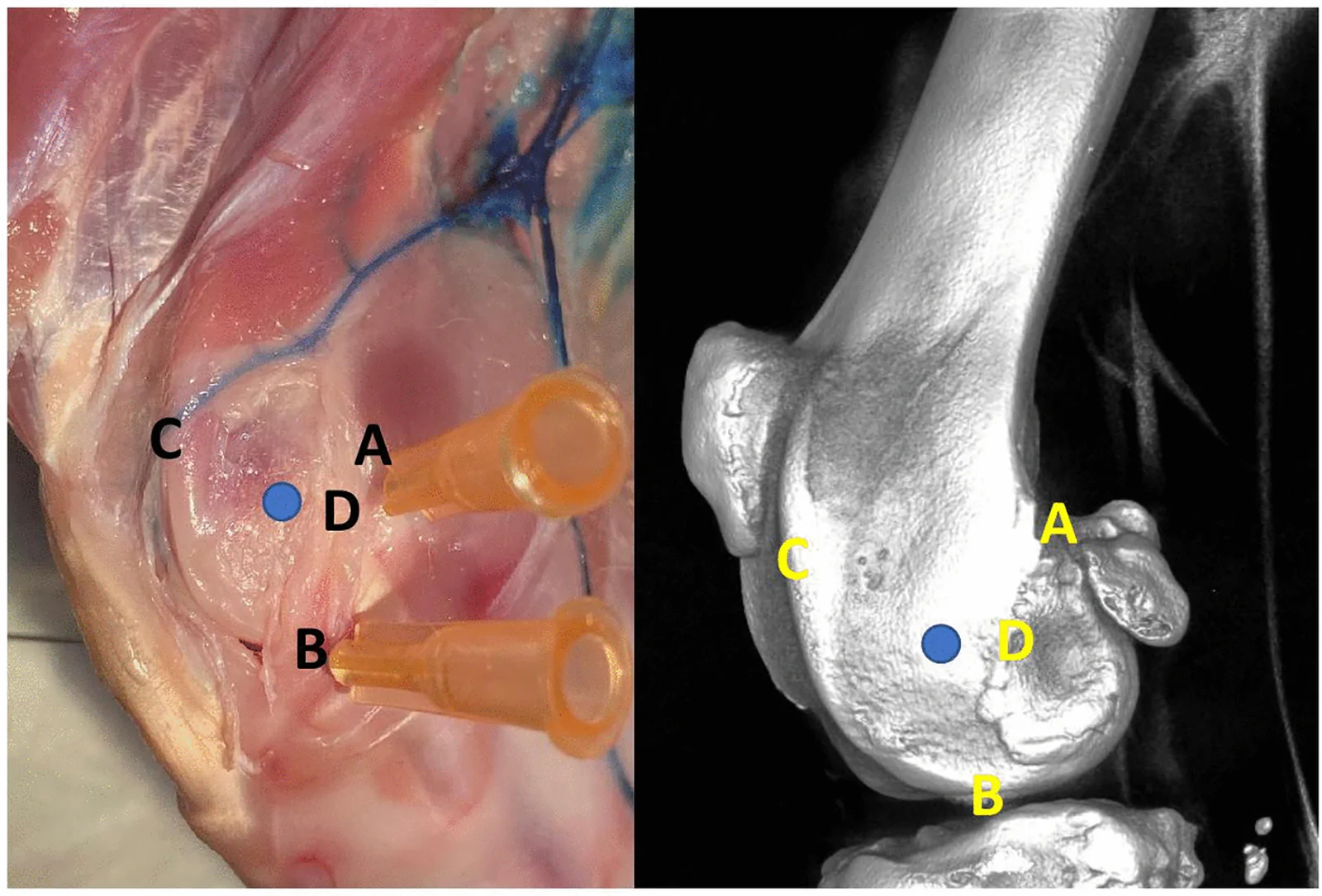
Left: Methylene blue infusion, Right: Contrast µCT. A: Caudal edge of distal femur at the level of medial fabella, B: Distal joint line, C: Medial trochlear ridge, D: Medial epicondyle.: Entry point of 4.5 mm pilot hole.

#### Surgical landmarks.

Useful bony landmarks for orientation and placement of the entry of the pilot drill hole include the caudal edge of the distal femur at the level of the medial fabella, the medial epicondyle at the origin of the medial collateral ligament, the proximal aspect of the medial trochlear ridge and the distal joint line, which may be located with direct palpation or sounding with a small gauge needle. Placement for the entry of the 4.5 mm pilot hole is within the triangular region bordered by these landmarks, immediately cranial to the medial epicondyle, on the mineralised physis, visible as a slightly raised ridge running cranial to caudal on the medial condyle, often present as a distinct demarcation in colour from red-to-purple proximally to whiter distally.

### In vivo animal model results

#### Animal numbers and defect allocation.

2040 individual distal condylar defects were created in 1020 rabbits between 2017 and 2022. Of these, 56 empty defects at 6 weeks and 72 empty defects at 12-week timepoints; and 50 autograft-filled defects at 6 weeks and 52 autograft-filled defects at 12-week timepoints were available for inclusion in the study.

#### Surgical time.

Exposure and harvest of iliac crest autograft took approximately 10–15 minutes while exposure and defect creation took approximately 5–10 minutes to complete bilaterally. Closure of the surgical site was generally completed within 5 minutes.

#### Complications.

Potential complications related to this model can result from anaesthetic, surgical or medical issues. Surgical complications can arise considering the anatomy and the small margin of error. This includes fracture that can be detected at the time of surgery and patella subluxation which can be observed following recovery. In our series of 2040 defects, fracture at the time of surgery was noted in 1 limb (0.05%), and patella subluxation was detected post-operatively in 3 limbs (0.15%). Anaesthetic death occurred in one animal (0.1%).

Surgical site inflammation of either the defect or autograft harvest site was occasionally noted, which was managed with additional NSAID and/or analgesia administration. The choice of medications given was at the discretion of the study veterinarian, based on the clinical parameters listed earlier. Wound dehiscence due to self-traumatisation was infrequent and was managed by either close observation until healing by second intention was complete or by induction of general anaesthesia and suturing of the wound.

#### Healing of distal femoral defects.

Representative µCT, PMMA and paraffin histology slices of empty defects at 6- and 12- weeks post-operatively are shown in Fig 6. In empty defects, the healing response of the tissue was primarily consigned to restoring some continuity of the cortex, rather than restoring bone within the metaphyseal cancellous region which remained empty. Below the surface, activity was present at the margins of the defect, initially forming some new woven bone at the periphery at week 6, before consolidating into a more refined trabecular structure continuous with the surrounding bone at week 12. Minimal bone was produced beyond the margin towards the centre of the defect, however, throughout the period of observation thus establishing the critical nature of the model.

**Fig 6:**
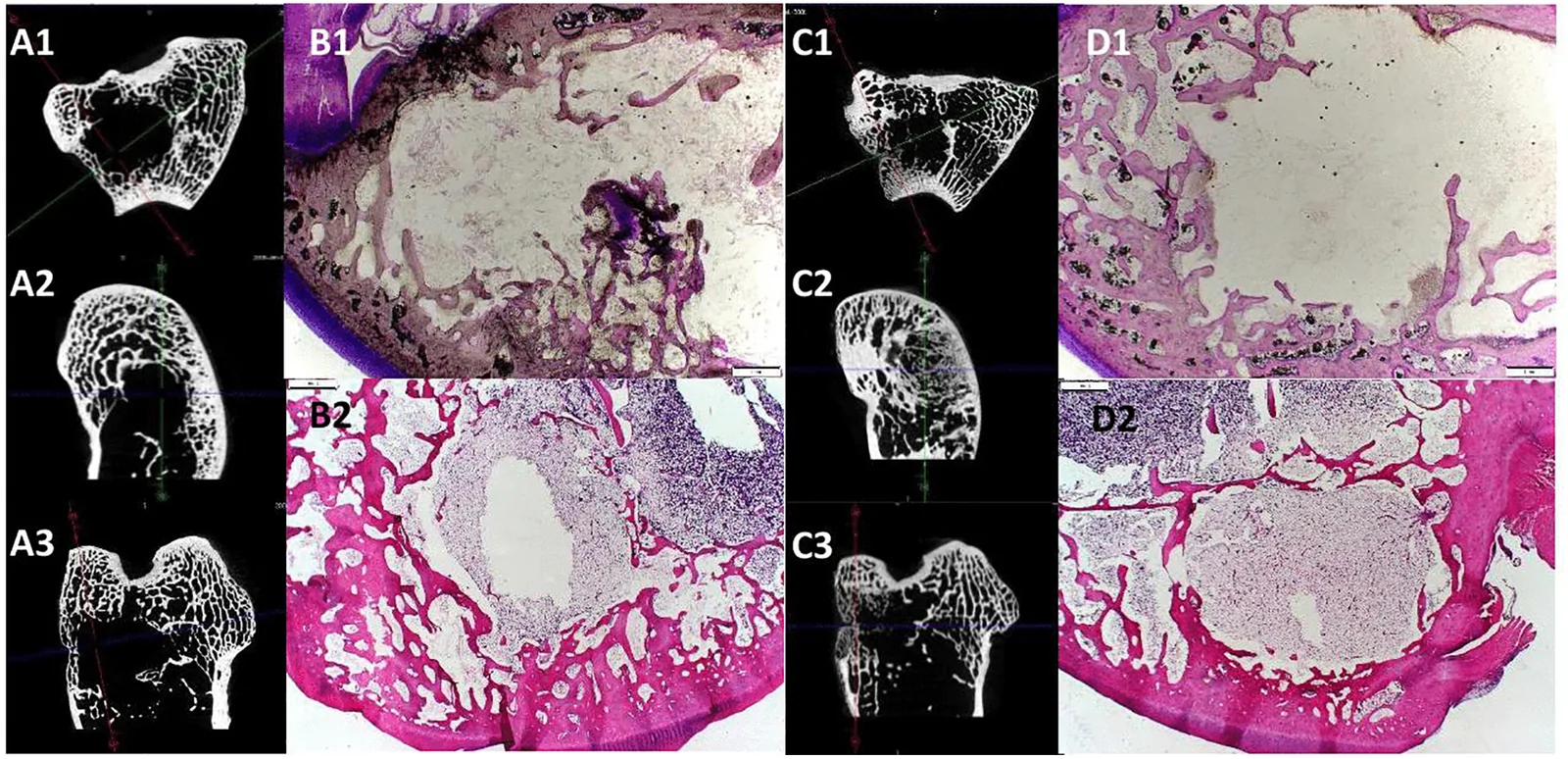
Bone healing response of an empty critical size defect in the rabbit distal femoral condyle 6 and 12 weeks post-operatively. A and B images: 6 weeks post-operatively. C and D images: 12 weeks post-operatively. A: µCT – 1: transverse plane, 2: sagittal plane, 3: coronal plane. B1: PMMA histology, methylene blue and fuchsin – sagittal plane, B2: Paraffin histology, H&E – sagittal plane. 2 x objective magnification. C: µCT – 1: transverse plane, 2: sagittal plane, 3: coronal plane. D1: PMMA histology, methylene blue and fuchsin – sagittal plane, D2: Paraffin histology, H&E – sagittal plane. 2 x objective magnification.

At a cellular level, some marrow elements similar in appearance to that of adjacent cancellous bone as well as fibrovascular tissue were present throughout the region of the defect at both 6 and 12-week timepoints. Numerous blood vessels and scant fibrous tissue was visible throughout the defect at week 6 and 12. Osteoblasts were observed populating the surface of the peripheral trabeculae at 6 weeks. At 12 weeks, fewer areas of osteoblasts were present. Similarly, whilst there was some osteoclastic activity at the periphery of the defect at week 6, at week 12, this was less apparent. Osteonecrosis was not observed in the peripheral trabeculae at either timepoint.

Healing of the surface progressed through bridging of the defect gap with trabeculae that became more confluent with the defect margin over time, eventually reforming a surface with an identifiable cortical shell and underlying cancellous trabecular architecture. Whilst the continuity of the surface with the surrounding bone can be partially restored at week 12, the anatomical contour of the surface was not restored, rather the surface took on a concave, sunken appearance. The cancellous bone within the defect created does not spontaneously reform in the empty defect condition thereby confirming the critical nature of the model.

Representative µCT, PMMA and paraffin histology slices in the sagittal plane of defects filled with corticocancellous autograft harvested from the iliac crest at 6- and 12-weeks post-operatively are shown in Fig 7. Corticocancellous autograft implanted into defects became incorporated into the surrounding cancellous architecture at week 12. The margins of these defects similarly appeared delineated and denser than the implanted or surrounding bone at week 6, whilst at week 12, a continuous, traversing trabecular structure existed. Similarly, the surface of the defect was restored at week 12, however in contrast to empty defects, this took place along the surface of the implanted graft material, which acted as a barrier to soft tissue ingress into the defect, maintaining a more anatomical contour.

**Fig 7:**
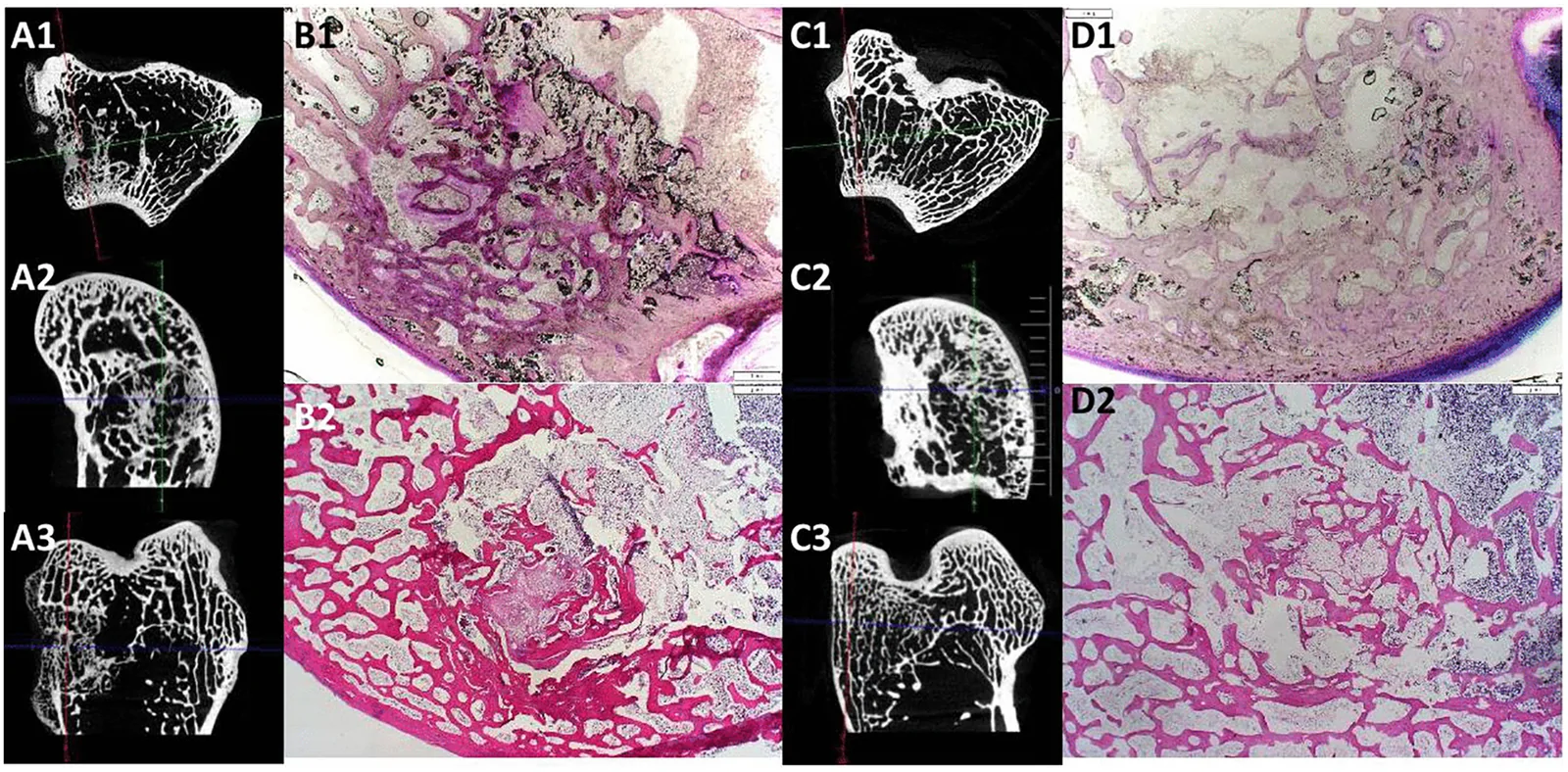
Bone healing response of a critical size defect in the rabbit distal femoral condyle filled with corticocancellous autograft at 6 and 12 weeks post-operatively. A and B images: 6 weeks post-operatively. C and D images: 12 weeks post-operatively. A: µCT – 1: transverse plane, 2: sagittal plane, 3: coronal plane. B1: PMMA histology, methylene blue and fuchsin – sagittal plane, B2: Paraffin histology, H&E – sagittal plane. 2 x objective magnification. C: µCT – 1: transverse plane, 2: sagittal plane, 3: coronal plane. D1: PMMA histology, methylene blue and fuchsin – sagittal plane, D2: Paraffin histology, H&E – sagittal plane 2 x objective magnification.

Autograft treated defects at 6 weeks post-operatively acellular autograft bone fragments were surrounded by some newly formed woven bone, fibrovascular tissue with small pockets of fibrous marrow tissue scattered throughout. There were regions of fibrous tissue conversion to new trabecular bone, evident with osteocyte-filled lacunae and osteoblasts present along the surface of the trabeculae. No evidence of infection or adverse reactions were noted histologically.

At 12 weeks, composition of the defect was similar to that of the surrounding cancellous bone of the distal femur. Much of the fibrovascular tissue had been replaced with a trabecular network composed of acellular bone fragments, new trabecular bone inhabited by osteocytes within lacunae and osteoblasts on the surface, and fibrous tissue organised in trabecular-like structure, with blood vessels and a larger volume of marrow components interspersed throughout. A normal bone remodelling cascade was observed typical of autograft healing.

## Discussion

We describe our experience with the distal femoral condylar defect model in cancellous bone in the skeletally mature rabbit. We have included an ex vivo characterisation of the local vascular anatomy, surgical technique, peri-operative husbandry considerations, potential complications with the model and typical healing responses of empty and autograft-filled defects to provide context for future studies of cancellous bone healing as well as for other research groups using this model. The aim of this manuscript was to provide a technical reference for the field and share our experiences using skeletally rabbits as an experimental model.

### Bone healing responses

The in vivo rabbit distal condylar defect model in skeletally mature animals allows evaluation of the bone healing response, as well as the inflammatory and immunological responses and material degradation processes that would not otherwise be possible in vitro or in silico [21]. Bone healing represents a complex, modulated biological response to injury involving genetic expression, cellular signalling and recruitment, ultimately resulting in regeneration of physiologically and mechanically functional tissue [26,27]. Several characteristic mechanisms by which bone healing occurs, or is supported are commonly considered, however there exists some variation in definition in the literature, often depending on the context in which it is used, e.g., biomaterial, cellular biology or clinical contexts. These include: osteoconduction, by which a scaffold for bone growth along a surface or within a porous structure is present; osteoinduction, referring to the stimulation of precursor cells to differentiate into tissue forming cells within the healing pathway by proteins; and osteogenesis, by which cells present may produce bone or differentiate into bone producing cells (i.e., including osteoblasts or precursors) [14,15,28,29]. These biological strategies can be evaluated in the distal femoral condylar defect model.

### Critical size defects

Critical size defects (CSD) are often used to experimentally examine the healing response of bone, to evaluate the effects of a device, bone graft or graft substitute in a wound that otherwise would not heal. A CSD is defined as “the smallest size intraosseous wound in a particular bone and species of animal that will not heal spontaneously during the lifetime of an animal” or “defects that have < 10% bony regeneration during the lifetime of the animal” [30]. A CSD is distinct from a non-union, in that, in the presence of favourable biology the defect will support healing by new bone formation [18]. Rabbit CSD models have been used extensively by many groups in biomedical research [31–35], however results between studies can be difficult to compare due to variables such as surgeon, surgical technique, defect size, animal age, breed, sex, and post-operative management. The use of other anatomical locations for similar defect models has also been reported including the cranium [36], long bones [37,38], tibial cortical window [39] and cancellous defects in the tibia and femur [40]. Healing rates between these sites, however, may not be directly comparable due to variation in structural, vascular, mechanical, and biological environments. The distal femoral condylar defect elegantly satisfies the requirements of an in vivo CSD model, in that it does not heal within the duration of the study, whilst the remaining cancellous and cortical structure of the distal femur is inherently mechanically stable [20] without the need for supplemental fixation. Animals tolerate the creation of defects in both femurs, allowing reduction of animal numbers to satisfy study sample requirements whilst considering the 3R’s principle [41]. Care should be taken regarding the extent of soft tissue exposure to minimize local tissue damage and disruption of local vasculature as well as to avoid patella subluxation which can compromise the animal’s ability to ambulate post-operatively.

Various sized distal femoral condylar defects have been used in rabbits throughout the biomedical literature, with diameters of 3 – 8 mm and depths ranging from 5 – 15 mm [35,42–46]. From a study design perspective, the smallest defect that enacts the biological response of a critical size defect whilst minimising animal morbidity would be ideal. The 6 mm diameter and 10 mm depth also provides an implantation site that allows a range of graft material sizes that are used in humans to be examined with this model. In our experience, although the 6 mm diameter, 10 mm depth defect functions as a critically sized cancellous bone defect and has been reported as a CSD by others [47–49], the cortical surface can partially reform in empty defects as well as autograft treated defects. This could be relevant to studies utilising shallower defect depths, as the reforming cortical shell would further reduce the depth of the defect throughout the duration of the study. The cancellous bone removed during the defect creation does not spontaneously heal, thereby providing confirmation of the critical size defect model.

Based on the histological appearance of the defects in this study, empty defects restore some marrow components by 6 weeks with bone remodelling activity at the periphery of the defects adjacent to the host bone. Bone remodelling was reduced at 12 weeks, and whilst the defects still demonstrated some osteoblastic and osteoclastic activity at the periphery, there was no attempt to bridge the cancellous region of the defect with new woven bone. In contrast, in autograft-filled defects, between acellular bone fragments, representing autogenous corticocancellous bone chips, fibrovascular tissue predominated, with some new trabecular bone and small regions of fibrous marrow tissue. Both osteoblastic and osteoclastic activity was present throughout the tissue at 6 weeks, whilst at 12 weeks. Whilst empty defects had not filled with bone but remained filled with marrow and some fibrovascular tissue at 12 weeks, the autograft-filled defects in this study exhibited a trabecular structure continuous with the surrounding bone of the metaphysis. Hence, empty defects functioned as CSDs, whilst the autograft-filled defects functioned as an effective positive control over the time course of the study. This is a technique paper with qualitative results. Quantitative measures such as histomorphometry and bone morphometry were not performed but would allow quantifiable evaluation of defect healing.

### Defect position and surgical approach

Meticulous soft-tissue dissection, preservation of local vasculature, avoiding the use of electrosurgery and applying saline irrigation during drilling are considered essential components of the method, minimising unnecessary pain and inflammation, whilst preserving the healing response necessary for the in vivo model. Accurate defect positioning is essential to maximise the available volume of cancellous bone for material placement, as well as avoid morbidity [50]. Animals tolerate the creation of defects in both femurs, allowing reduction of animal numbers to satisfy study sample requirements whilst considering the 3R’s principle [41].

The margin for defect placement completely within the cancellous architecture of the condyles is small. The typical craniocaudal width of the distal femoral condyle is approximately 10 –11 mm in the rabbit typically used in our studies, leaving only 4–5 mm of bone cranial and caudal to a 6 mm defect, if drilling is centred and progresses precisely in a transverse direction. Drilling too proximally may result in communication of the defect with the intramedullary canal, resulting in bleeding from the defect that can be difficult to control, whilst a cranial or distal location risks intra-articular communication, which would result in synovial fluid communication and increased animal morbidity, posing a welfare concern as well as confound the biological response of an implanted graft material under evaluation.

The surgical approach we used considered the ideal placement of the defect in the cancellous bone bed, as well as respecting the local soft-tissue and vascular envelope. In our experience, minimal haemorrhage was encountered during the approach, which has been refined with a knowledge of the vascular anatomy to preserve the delicate overlying tissues through limited tissue dissection, undermining and reflection of the soft-tissue planes, allowing precise re-apposition of these structures over the defect. We have generally found electrosurgery overly traumatic to the delicate tissues and not useful in this small animal model and prefer to control haemostasis through direct pressure, preserving the soft-tissue envelope as much as possible. Meticulous haemostasis is essential during test material implantation as the pressure of persistent blood flow from the defect is sufficient to dislodge some materials, particularly those that have not yet completed phase transition from liquid to solid, which can result in an incomplete filling of the defect.

### Technical considerations

The surface of the medial condyle of the distal femur of the rabbit is sloped relative to the sagittal plane, such that the drill bit tends to skive cranially prior to engagement of the cutting elements in the bone, particularly with application of fluid irrigation, which acts as a lubricant [51]. Skiving of the drill may be minimised by use of restrained axial force, low drilling speeds and first engaging the tip of the bit at a perpendicular angle to the cortical surface, then redirecting the trajectory towards the centre of the distal femur. 3-fluted drill bits have been shown to have greater stiffness than bending than 2-fluted bits, improving angled targeting [52]. We find the pyramidal tip of the Surgibit® (OI, Collaroy, NSW) particularly useful for accurate positioning as the cutting edges converge upon a single point, as opposed to the linear chisel edge of a traditional 2-fluted bit which can easily skive and effect defect placement. Finally, grasping the shank of the bit close to the termination of the flutes, rather than at the end of the shank shortens the working length of the bit, increasing the bending stiffness of the bit and improves targeting and facilitates accurate and consistent placement of the defects, avoiding skiving in an anterior and distal direction, towards the joint space.

Step-drilling with a 4.5 mm bit followed by a 6 mm bit and application of external irrigation are performed to minimise the thermal impact of the drilling procedure on the biology of the defect [53,54]. Thermal necrosis is a function of peak exposure and duration of exposure, and it has been shown that the regenerative capacity of bone is reduced at exposures to temperatures as low as 44 °C for one minute [55]. Variation in drilling forces produced by surgeons, drilling speeds, drill bit geometries and attention to irrigation during drilling could influence the healing of defects in this model if care is not taken to maintain consistency in drilling parameters. Lavage of the surgical site following drilling is also performed to remove swarf from the soft tissues, which might otherwise cause irritation during convalescence.

### Complications and management considerations

Anaesthesia of rabbits may be complicated by hypothermia, cardiovascular effects of drugs and gastrointestinal stasis [23,56,57]. These complications may be mitigated by a thorough pre-operative veterinary exam facilitating enrolment of healthy animals into the study, and careful anaesthetic administration and monitoring. A very low incidence of anaesthetic-related complications occurred in our cohort, supporting the use of the anaesthetic protocol.

No complications influencing study outcomes, or necessitating removal of the animal from the study were observed in the post-operative period.

Close post-operative monitoring of animals is essential to maintain welfare. Objective pain scores such as the grimace scale may be useful in the post-operative assessment of the animals, in addition to monitoring of appetite, thirst, urination, defecation and movement [58].

Healing of superficial tissues may be complicated by wound breakdown and superficial wound infection, reported previously [23]. With diligent post-operative monitoring, these complications can be effectively controlled without impact on study outcomes. *Pasteurella multocida* and *staphylococcus* species are the most commonly implicated bacterial organisms in surgical site infection of rabbits, however streptococcus and various anaerobic bacteria may also be implicated [59]. *Pasteurella* species are of particular concern in laboratory rabbits due to this endemic species’ propensity to cause multisystemic disease in some individuals [60,61] whilst producing no symptoms in others [62]. Surgical treatments used in veterinary clinical practice for more severe infections may include establishing open drainage, lavage with balanced isotonic fluid and appropriate antibiotic therapy or en bloc excision of infected material and surrounding fibrous pseudo-capsule in compartmentalised abscessation in which simple drainage is not effective [59]. In the research setting, treatment choices must be chosen to reduce additional stress and morbidity to the animals from repeated handling, which may impact welfare [63]. In selecting antibiotic therapy, we find the intramuscular administration of procaine penicillin every 48 hours most effectively satisfy these parameters [64].

Lateral patella luxation following a medial parapatellar approach has been observed by others [65]. Symptoms of patella luxation include lameness with external rotation of the foot and palpable subluxation of the patella, lateral to the lateral trochlear ridge. These authors attributed this complication to misadventure during post-operative housing and recommend routine singular housing for 2–3 weeks followed by paired housing for 3–4 weeks. Whilst reducing post-operative activities may reduce forces on the surgical site closure, this must be balanced with institutional ethical housing requirements which may require group housing. In addition, excessive exposure of the surgical site can also adversely affect patella stability. At our institution, animals requiring intensive observation or removal from group interactions as part of a veterinary treatment regime may undergo isolation in single pen housing for the duration of treatment, after which resocialisation according to preoperative schedules may be attempted, if sufficient time remains within the study. In veterinary clinical practice, medial patella luxation has been reported in a small number of rabbits, with both developmental and traumatic causes suspected [66–68]. Surgical treatment of the condition has been described; however, medical management is frequently prescribed and may be an appropriate option in cases of adequate function with minimal pain [66–68]. In addition to functional lameness, a degree of pain, synovitis, trochlear cartilage erosion and periarticular osteophyte formation have been observed clinically and at necropsy secondary to medial patella luxation in rabbits [65–68]. This is unlikely to affect the results of a bone defect model, however, would bear consideration in an articular healing model. Similar post-operative luxation has been reported in sheep undergoing medial parapatellar incisions [69]. A limited medial approach has been described to reduce risk of post-operative luxation in the sheep [70], however, due to the location of the defect and need for sufficient soft-tissue clearance to allow entry of the drill bits to the bone in this model, preservation of these structures would not be feasible in this rabbit model.

We observed a very low incidence of complications, which we attribute to the pre- peri- and post-operative management considerations detailed within. Reporting of complete clinical records is beyond the scope of this study, however, many of the commonly reported concerns may be readily managed with diligent post-operative monitoring and animal husbandry.

## Conclusion

ln vivo models provide invaluable, irreplaceable preclinical data informing the in vivo response; however, such high – impact studies must have a sound ethical basis and be conducted with animal welfare and scientific rigour at the forefront of study design and execution. A multitude of factors including the peri-operative animal husbandry, surgical technique, defect location and material placement all work in concert to influence the healing response. We share our experience with the local anatomy, surgical technique, peri-operative husbandry, and the typical healing responses of empty and autograft – filled defects are reported here to provide context for future studies of bone healing. The distal femur defect model is a valuable component in the context of pre-clinical data for product development as well as regulatory submissions for graft materials not related to the intrinsic stability of the bone.

## Abbreviations

FDA: Food and Drug Administration
NZW: New Zealand White
CT: Computed Tomography
ACEC: Animal Care and Ethics Committee
PMMA: Poly methyl methacrylate
SPO2%: haemoglobin oxygen saturation percentage
ETCO_2_: end-tidal carbon dioxide percentage
EDTA: Ethylenediaminetetraacetic acid
NSAID: Non-steroidal anti-inflammatory drug
H&E: Haematoxylin & eosin
CSD: Critical Size Defect
